# Editor’s introduction to this issue (G&I 20:4, 2022)

**DOI:** 10.5808/gi.20.4.e1

**Published:** 2022-12-30

**Authors:** Taesung Park

**Affiliations:** Department of Statistics, Seoul National University, Seoul 08826, Korea

In this issue, there are two review articles, eight original articles, and one application note. Three of these articles are related to genetic association studies. The first review article, by J. Ott (Rockefeller University, New York, USA) and T. Park (Seoul National University, Seoul, Korea), is about frequent pattern mining (FPM) analysis. FPM has been widely applied to genetic problems, specifically to the combined association of two genotypes at different DNA variants with diseases. FPM methods have the ability to select genotype patterns that are distinct between cases and controls. In particular, FPM has been quite effective for gene-gene interaction (GGI) analysis. For example, the multifactor dimensionality reduction (MDR) method is a representative FPM method for detecting GGIs. Since its first introduction, MDR has been popularly used for GGI analyses. One of the challenges in FPM is to assess the statistical significance of these selected patterns, which requires a heavy computational burden and suffers from the issue of multiple comparisons. This review discussed these issues in a reasonable way.

The second article, by M. Park’s group (Eulji University, Daejeon, Korea), proposes the multi-level polar Lasso (MP-Lasso) chart, which is a visualization tool to summarize the results of group Lasso and sparse group Lasso. In large-scale genetic association studies a set of important markers should be selected simultaneously. In these cases, penalized regression has been widely used in genome-wide association studies (GWAS). Among penalized regression models, Lasso effectively selects some important markers for the model by shrinking unimportant markers toward zero. Group Lasso and sparse group Lasso have been proposed to take into account the structures of groups, such as genes and pathways. Group Lasso selects some important groups of markers from the model and eliminates unimportant groups, thereby ensuring sparsity at the level of pre-defined groups. As in group Lasso, sparse group Lasso performs group selection, but also individual selection as well. Although these sparse methods are useful for high-dimensional genetic studies, interpreting the results with many groups and coefficients is not easy. Trace plots of the regression coefficients are commonly used to present Lasso results. However, studies that systematically visualize group information are rare. In this article, the authors propose an MP-Lasso chart that can effectively express the results of group Lasso and sparse group Lasso analyses. An R package for drawing MP-Lasso charts was developed. Through real data applications, the authors demonstrate the usefulness of the MP-Lasso charting package effectively by successfully visualizing the results of Lasso, group Lasso, and sparse group Lasso. The visualization of high-dimensional data is quite challenging. One of the advantages of MP-Lasso is that it can be applicable to any type of omics data.

The third article, by W. Lee’s group (Medical Genomics R&D, Seoul, Korea), is about the meta-analysis of GWAS. Meta-analysis has become a standard method after individual GWAS, because it takes advantage of a large sample size by combining multiple studies. While many packages for meta-analyses have been developed to discover genetic variants, but not many currently accessible packages consider between-study heterogeneity well. Most packages allow random-effects models for handling between-study heterogeneity. However, determining whether to include random effects is not straightforward. The authors propose the Beta-Meta software, which is Python-based and can easily conduct a meta-analysis by automatically selecting between a fixed-effects and a random-effects model based on heterogeneity. Beta-Meta has many advantages, such as flexible input data manipulation and a step-by-step meta-analysis of GWAS for each association. In particular, Beta-Meta performs heterogeneity testing first, with two different calculations of the effect size and the p-value based on heterogeneity, and the Benjamini-Hochberg p-value adjustment. The authors elaborate on these points and illustrate them with real data examples. I expect that Beta-Meta will become a powerful tool for meta-analyses.

